# Bibliographical

**Published:** 1893-02

**Authors:** 


### Bibliographical.

A SERIES OF QUESTIONS AND ANSWERS FOR DENTAL STUDENTS. By Prof. F. J. S. Gorgas.

The third part of this Series of Questions and Answers has just come to hand. What we said of the first two parts will apply to this. The subjects, upon which the questions and answers are based are those embraced in the curriculum of the senior year of the Dental Department of the University of Maryland, and as an elementary work upon these subjects it is very good indeed, and will be quite an aid to all students who will have the opportunity, or be disposed to consult it. As much is presented in this volume on the various subjects embraced as could be condensed in so small a space. It is well worthy of commendation to the profession and to students.

It is to be hoped Dr. Gorgas will be able to give a little closer attention to the proof when a second edition is issued.

The Transactions of the California State Dental Society for 1892 has just come to hand. It makes a volume of 200 pages, full of fresh and interesting matter, in the form of papers and discussions. For those who could not attend the meeting, the next best thing, is to read the record of the proceedings and take in the many new and instructive thoughts and suggestions to be found there.

A copy can be procured by writing to the Corresponding Secretary, Dr. Chas. E. Post, 302 Stockton St., San Francisco. It is one of the State Dental Societies so fortunate as to be able to publish the full proceedings of its annual meetings. Every State Dental Society should aim to do the same thing, and there are but few, indeed, who could not easily accomplish it if they possessed the interest in their work they ought to have.

